# Pathogen-host adhesion between SARS-CoV-2 spike proteins from different variants and human ACE2 studied at single-molecule and single-cell levels

**DOI:** 10.1080/22221751.2022.2128887

**Published:** 2022-11-04

**Authors:** Xiaoxu Zhang, Bixia Hong, Peng Wei, Pengfei Pei, Haifeng Xu, Long Chen, Yigang Tong, Jialin Chen, Shi-Zhong Luo, Huahao Fan, Chengzhi He

**Affiliations:** a Beijing Advanced Innovation Center for Soft Matter Science and Engineering, College of Life Science and Technology, Beijing University of Chemical Technology, Beijing, People’s Republic of China; b School of Traditional Chinese Medicine, Beijing University of Chinese Medicine, Beijing, People’s Republic of China; c Beijing Laboratory of Biomedical Materials, College of Materials Science and Engineering, Beijing University of Chemical Technology, Beijing, People’s Republic of China; d State Key Laboratory for Functions and Applications of Medicinal Plants, Guizhou Medical University, Guiyang, People’s Republic of China; e The Key Laboratory of Chemistry for Natural Products of Guizhou Province and Chinese Academy of Sciences, Guiyang, People’s Republic of China

**Keywords:** SARS-CoV-2, pathogen-host adhesion, force spectroscopy, neutralizing antibody

## Abstract

The binding of the receptor binding domain (RBD) of severe acute respiratory syndrome coronavirus 2 (SARS-CoV-2) spike protein onto human angiotensin-converting enzyme 2 (ACE2) is considered as the first step for the virus to adhere onto the host cells during the infection. Here, we investigated the adhesion of spike proteins from different variants and ACE2 using single-molecule and single-cell force spectroscopy. We found that the unbinding force and binding probability of the spike protein from Delta variant to the ACE2 were the highest among the variants tested in our study at both single-molecule and single-cell levels. As the most popular variants, the Omicron variants have slightly higher unbinding force to the ACE2 than wild type. Molecular dynamics simulation showed that ACE2-RBD (Omicron BA.1) complex is destabilized by the E484A and Y505H mutations and stabilized by S477N and N501Y mutations, when compared with Delta variant. In addition, a neutralizing antibody, produced by immunization with wild type spike protein, could effectively inhibit the binding of spike proteins from wild type, Delta and Omicron variants (BA.1 and BA.5) onto ACE2. Our results provide new insight for the molecular mechanism of the adhesive interactions between spike protein and ACE2 and suggest that effective monoclonal antibody can be prepared using wild type spike protein against different variants.

## Introduction

**Figure 1.**
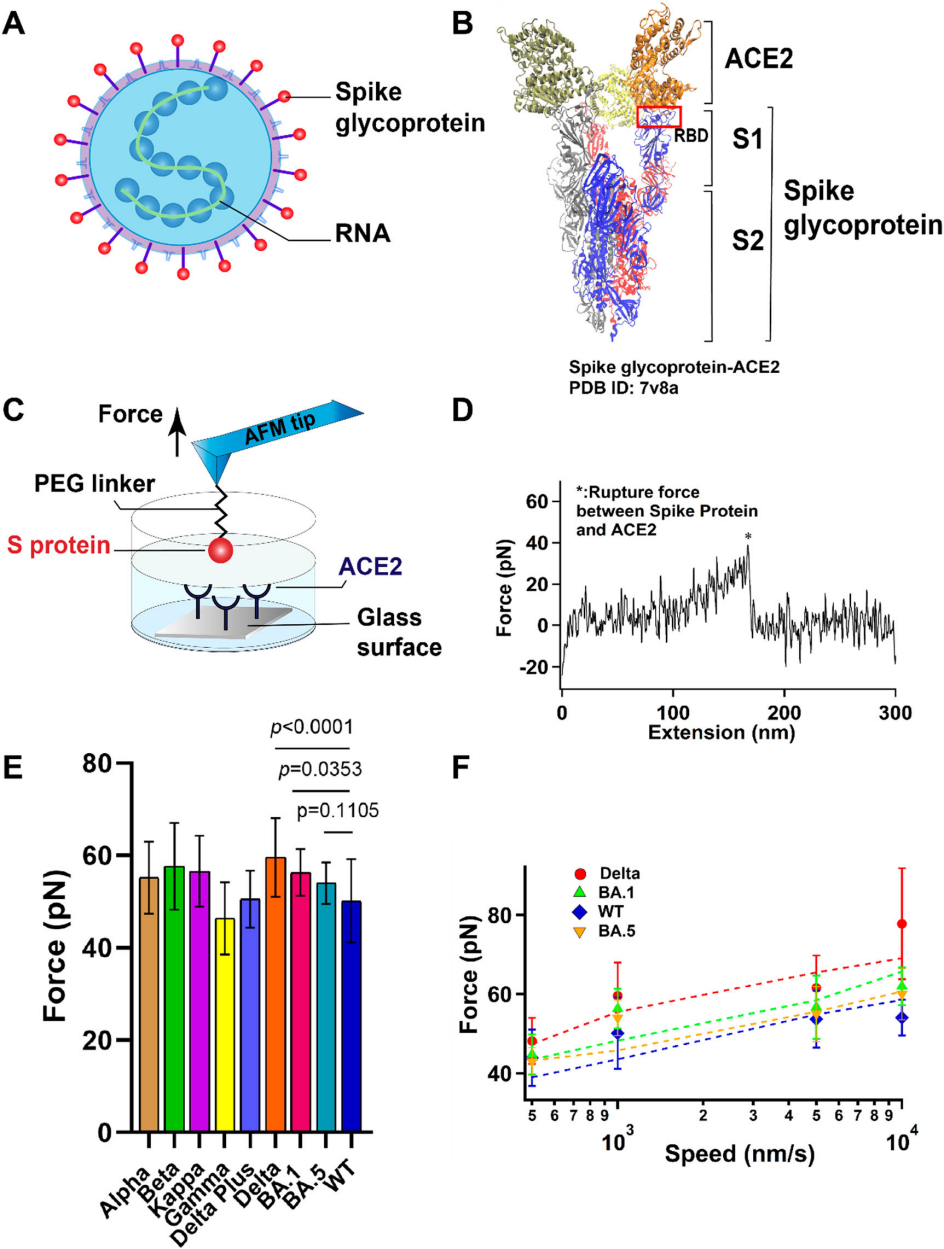
The interaction between S protein and ACE2 was studied by SMFS. (A) Schematic diagram of SARS-COV-2 particle, an enveloped ssRNA virus expressing spike glycoprotein (S) on its surface. (B) Crystal structure of Delta variant (B.1.617.2) in complex with three ACE2. S is divided into N-terminal S1 and C-terminal S2 domains. RBD, binding with ACE2, was located at the C-terminal of the S1 sub-domain. (C) Schematic of measurement of adhesive interaction between S protein and ACE2 using SMFS. The purified S protein and ACE2 were attached to probe and substrate, respectively. (D) A representative force-extension curve showing specific adhesive event labelled by star. (E) The adhesive forces between S proteins from different variants and ACE2. (F) Pulling speed dependency of adhesive forces and Monte Carlo simulations for extracting the kinetic parameter. Solid dots represent experimental data and dotted lines represent simulated results.

Severe acute respiratory syndrome coronavirus 2 (SARS-CoV-2) has rapidly spread to the entire world and become a devastating pandemic [1–4]. As the SARS-CoV-2 is an enveloped, positive-stranded RNA virus, mutation with increased infectivity and transmission is likely to happen during the evolution [5,6]. SARS-CoV-2 infects human host cells by an initial adhesive interaction of its receptor-binding domain (RBD) located in the C-terminal of the S1 subunit of spike protein (S protein) and angiotensin converting enzyme 2 (ACE2), the receptor on human cells, as shown in Figure 1(A,B) [2,7]. The spike protein is a large type I transmembrane protein containing two subunits, the N-terminal S1 and C-terminal S2 domains, and S1 comprises the N-terminal domain (NTD) and RBD. S2 contains basic elements needed for the membrane fusion, it could be cleaved by host proteases, and leading to the activation of the glycoprotein that undergoes extensive irreversible conformational changes facilitating the membrane fusion process [2,8]. Recently, a number of mutations were identified on the S protein in the SARS-CoV-2 variants, which have been reported with different infectivity and pathogenicity. In particular, a N501Y mutation in the RBD of S protein involve in Alpha (B.1.1.7), Beta (B.1.351), and Gamma (P1) has an increased affinity to ACE2 [9,10]. Meanwhile, the K417N/T and E484K mutations in the RBD of Beta (B.1.351) and Gamma (P1) may result in conformational changes [1]. The Delta variant (B.1.617.2) harbours L452R and T478K mutations in the RBD, which could enhance the stability of S protein and the affinity to ACE2 [1,11,12]. Compared with Delta variant, Omicron variants contain more mutations, among which there are 8 to 10 residues located on the interface of the S protein and ACE2. In the past two years, the Delta and Omicron variants have become two of the most popular variants among all the variants of concern (VOCs) [13,14]. The Omicron variant, in particular, spread rapidly and replaced other variants as the dominant strain [15,16]. Currently, it is believed that the Omicron variant is more infectious but less pathogenic than Delta variant [3,17].

The interaction between SARS-CoV-2 S protein and ACE2 has been studied using various methods such as Cryo-EM, X-ray, molecular dynamics simulation, surface plasmon resonance (SPR), flow cytometry, and force spectroscopy [2,4,6,17–23]. Here we investigated the adhesive interactions between S protein from different variants and ACE2 using atomic force microscopy-based single molecular force spectroscopy (SMFS) and Fluid microscopy-based single cellular force spectroscopy (SCFS). Atomic force microscopy (AFM)-based single-molecule force spectroscopy (SMFS) has greatly enriched our understanding of intramolecular and intermolecular interactions at the micro level. AFM probe tips are usually only a few tens of nanometres long and allow manipulation of individual molecules. Through chemical modifications and carefully designed experimental systems, we have improved the probability of capturing individual molecular events and precisely controlled the direction of force application. The mechanical strength of adhesion of ACE2 and RBD at the level of a single living cell was studied by SCFS, mimicking the adhesion behaviour of ACE2 and RBD in physiological environment. Molecular dynamics simulation was also used to investigate the binding mechanism for ACE2 and RBD from Delta and Omicron variants. We found that certain mutations of S proteins at the interface of RBD domain and ACE2 can stabilize and destabilize the complex, which complicates the impact of the mutations on the adhesive interactions. In addition, we evaluated the neutralization efficiency of a monoclonal antibody (mAb) obtained by immunization of mouse with the wild type (WT) RBD. We observed that the addition of mAb resulted in significant reduction of binding onto ACE2 for the wild type, Delta, and Omicron variants.

## Results and discussions

### Single-molecule adhesive interactions between S proteins and ACE2

Single-molecule force spectroscopy (SMFS) has become a powerful tool to characterize the mechanical property of macromolecules [24] and the protein-protein interactions [2,6,19,20] at single-molecule level. Here we use AFM based SMFS to measure the mechanical unbinding force between ACE2 and S proteins from nine different variants. By comparing the mutant amino acids of each Omicron sub-lineage variants, we selected BA.1 and BA.5 as Omicron test samples for subsequent experiments for both nearly contain all the amido acid mutants. ACE2 protein and all the S proteins were purchased from ACRObiosystems. All the S proteins were recombinant trimer proteins expressed from human 293 cells (HEK 293) with proline substitutions (F817P, A892P, A899P, A942P, K986P, V987P) and alanine substitutions (R683A and R685A) for the purpose of stabilizing the trimeric state and abolishing the furin site, respectively. We covalently attached purified ACE2 and S proteins to AFM probe and glass substrate, respectively, using a 20 kDa NHS-PEG-NHS linker (Figure 1(C)). The probe was approaching to and retracting from the substate at a constant speed of 1 μm/s in PBS buffer and at room temperature. A typical relationship between force and extension is shown in Figure 1(D). The force-extension curves (FECs) were fitted with the worm-like chain (WLC) model using the persistence length of 0.4 nm for the PEG linker. The single force-rupture events correspond to the mechanical unbinding of S protein and ACE2. Representative histograms of rupture force distribution for the interaction between ACE2 and different S proteins are shown in Figure S1 and the mutations of different variants are listed in Table S1. The unbinding forces of ACE2 and S proteins are summarized in Figure 1(E) and all the unbinding forces are in the range from ∼40 to 60 pN, which is in agreement with previous studies [2,6,19,20]. S proteins from all variants except the Gamma variant had greater unbinding forces to ACE2 than the wild type, although the difference between different variants is small. Among these variants, the Delta variant has the highest unbinding force to ACE2, which can reach up to 60 ± 9 pN (*n* = 157).

To extract the kinetics of the unbinding process, we conducted the dynamic force spectroscopy experiments at various pulling rates on S proteins. Figure 1(F) illustrates the trend of the binding force between the variant S proteins of interest, Omicron (BA.1 and BA.5), Delta, WT, and ACE2, as a function of the stretching rate. The force-speed relationship curves of Alpha, Beta, Kappa, Gamma, Delta Plus, and ACE2 were shown in Figure S2. According to the Bell-Evans model and Monte Carlo simulation [25], the dissociation rate constant *k*_0_ in the absence of a pulling force and the distance from native state to transition state Δ*x*_β_ were estimated as shown in Table 1. Our results showed that all the S proteins have similar Δ*x*_β_ and dissociation rate constant, suggesting that they have similar unbinding pathway and mechanical stability, although WT S protein-ACE2 is the least stable. Among those variants, the Delta has a higher binding force, and this stronger adhesion between the Delta variant and the ACE2 may contribute to the stronger cell invasion ability and high transmissibility [26] of the Delta variant compared to the other variants. We found the Omicron sub-lineages BA.5 has the lowest *k*_0_, indicating its higher affinity interaction although its binding force is not the highest.

**Table 1.** Kinetics extracted from dynamic force spectroscopy experiments.

S Protein-ACE2	*k*_0_ (s^−1^)	△*x*_β_ (nm)
WT S Protein-ACE2	0.075	0.71
Delta S protein-ACE2	0.026	0.68
BA.1 S protein-ACE2	0.029	0.73
BA.5 S Protein-ACE2	0.023	0.74
Alpha S Protein-ACE2	0.034	0.76
Beta S Protein-ACE2	0.034	0.73
Kappa S Protein-ACE2	0.033	0.74
Gamma S Protein-ACE2	0.045	0.77
Delta Plus S Protein-ACE2	0.039	0.78

### Single-cell adhesion between S proteins and cells

Force spectroscopy can not only be used at single-molecule level, but it can be expanded to single-cell level as well [2,20,27]. AFM is a useful tool to conduce single-cell force spectroscopy (SCFS) to characterize the interaction between a single cell and a surface as it has a broad range of measurable forces from pN to nN [28–30]. SCFS has previously been used in a variety of applications, such as studying the interactions between cells and substrate materials or hydrogels and mechanisms of adhesion between cells and extracellular matrix [28,31,32]. To further study the interaction between S proteins from different variants and ACE2 at cell level, purified S proteins and Vero cells with high expression of ACE2 were studied using SCFS. HEK293T cells were used as control cells because ACE2 protein is less expressed on the HEK293T cell surface. In addition, BSA-coated and blank glass substrates were chosen as control surfaces for cell adhesion.

**Figure 2.**
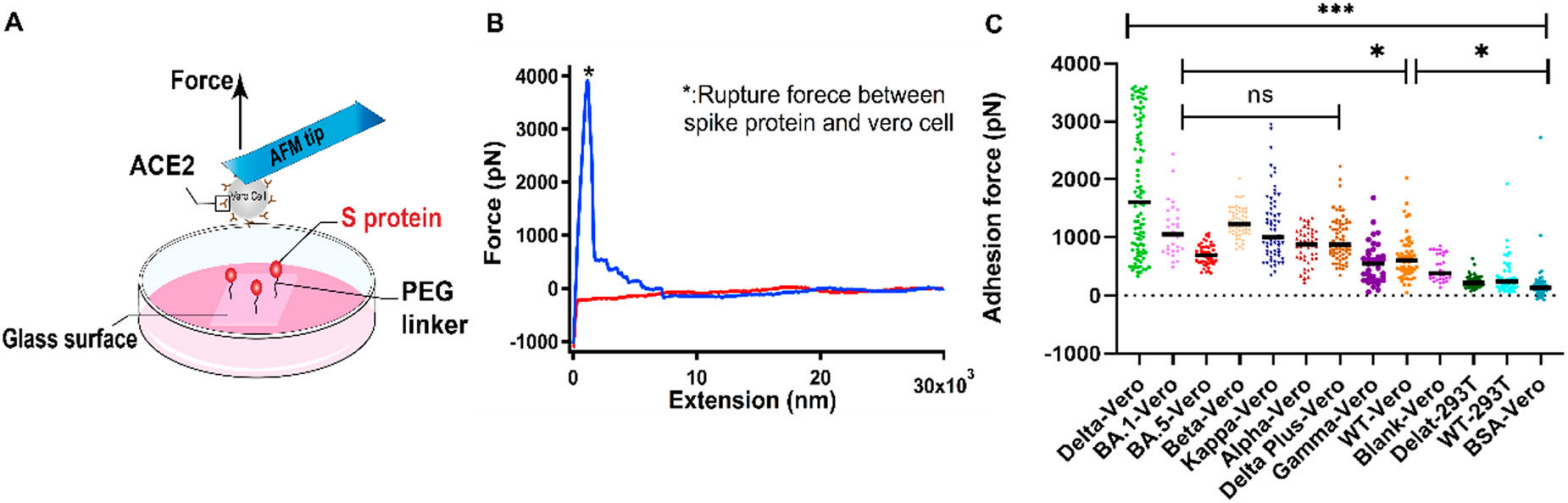
Experimental results of SCFS. (A) Schematic of SCFS experiments. (B) A representative force-extension curve of the detachment of Vero cell from S protein coated surface. (C) Adhesion force between cells and S proteins from different variants.

In our experimental setup, the attachment of a single cell to the tip of cantilever was achieved by adding a negative pressure of 50 mbar from a pump, and the probe is a micropipette with an aperture of 4 μm. The S proteins were immobilized on glass substrate using 20 kDa NHS-PEG-NHS linker the same way as the SMFS experiments. The cell-attached probe approached the substrate at a rate of 2 μm/s and contacted with the substrate for 5 s allowing the ACE2 on the cells to establish stable adhesion to S protein coated substrate, then retracted at the same rate to 30 μm (Figure 2(A)). When the cell is retracted from the S proteins coated surface, the adhesion force is precisely measured. A typical FECs were shown in Figure 2(B) and their maximum adhesion force was recorded. The rupture events occurred at the highest force correspond to the detachment of the bulk of cell from substrate.

The adhesion forces between cells and different surfaces were summarized in Figure 2(C). The S proteins from all the variants including wild type showed higher adhesion forces than the control groups, indicating that the S proteins indeed interact with ACE2, which is consistent with the results from previous studies [2,18]. Moreover, the results of SCFS is in agreement with our SMFS. The Delta variant showed the highest adhesion force among all the variants. No significant differences in the adhesion force were found for the Omicron variant compared with most other variants, although it is higher than wild type. Due to the multiple interactions of ACE2 on cells and the S proteins on the substrate, the adhesion force quantified by SCFS (nN level) is much higher than the single-molecule unbinding force of S protein and ACE2 (pN level). Among those variants, Omicron has been reported as the variant with the highest number of mutations and spread rapidly to become globally dominant variants [15]. In our SCFS experiments, we found, however, the binding force between Omicron (both for Omicron sub-lineages BA.1 and BA.5), similar to the SMFS, is not the highest, even lower than the Alpha and Kappa for the BA.5. So, we supposed that the high transmissibility may be is unrelated to the strength to the ACE2. The more mutations on spike protein maybe refer to a degree of immune escape [15].

### Inhibitory effect of neutralizing antibody on S proteins

**Figure 3.**
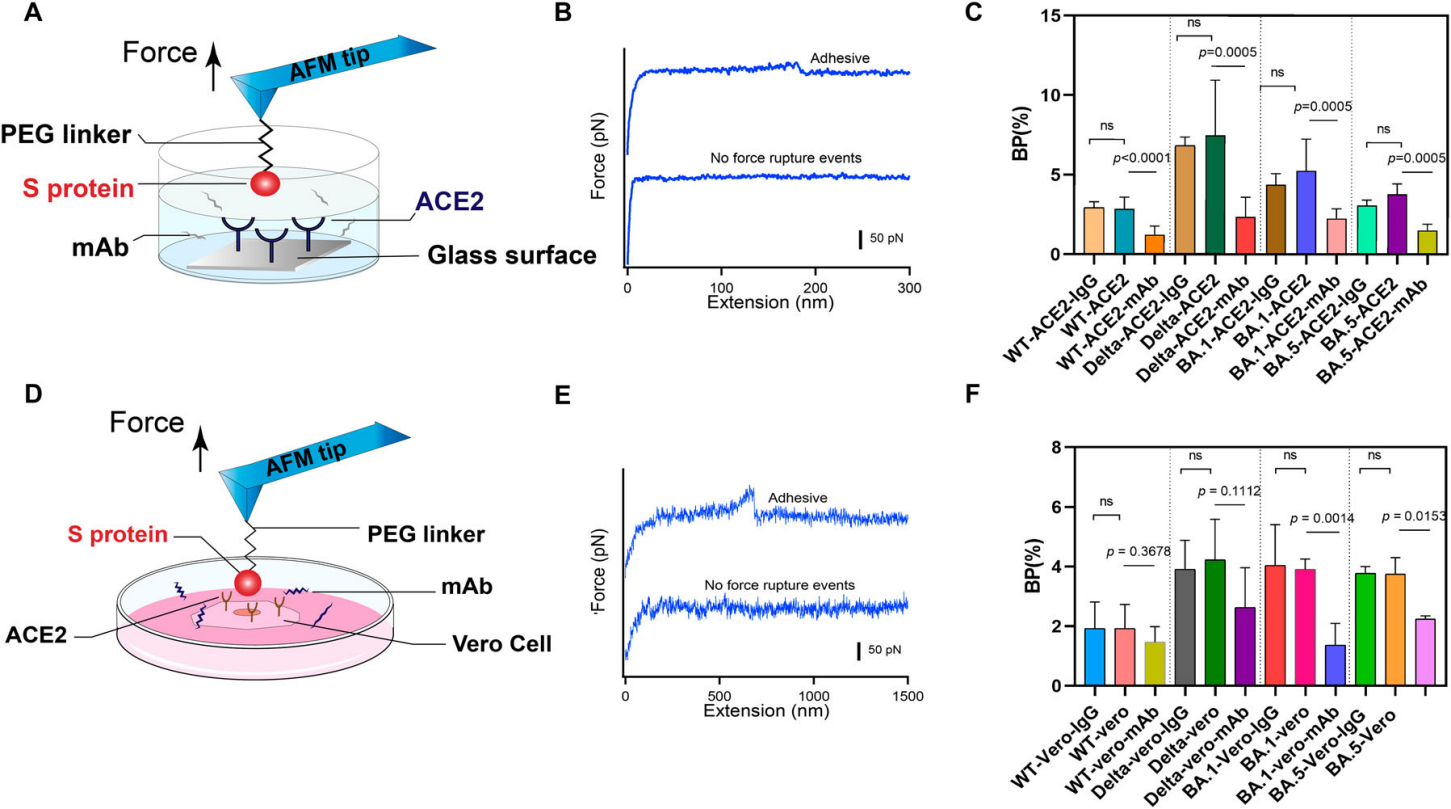
Anti-binding effects of mAb on S protein binding. (A) Efficiency of mAb is evaluated by measuring the BP of the interaction between the S proteins and ACE2 on model surface before and after incubation of the mAb. (B) Force-extension cures showing either nonadhesive and specific adhesive curves on model surface. (C) Histograms of binding probabilities (BP) between purified ACE2 and S proteins (WT, Delta and Omicron (BA.1 and BA.5)). (D) Efficiency of mAb is evaluated at the cellular level. (E) Force-extension cures measured on Vero cells. (F) Histograms of binding probabilities (BP) between Vero cells and S proteins (WT, Delta and Omicron (BA.1 and BA.5)). Experiments with isotype control antibody IgG1 and without addition of mAb were designed as control.

The SARS-CoV-2 invasion of human host cells is mediated by an interaction between the receptor binding domain on the S1 subunit of the glycoprotein anchored on the virus surface and human angiotensin-converting enzyme (hACE2) [6]. The potent neutralizing antibodies (NAbs), directed to the RBD, have been administered prophylactically after exposure to infectious virus, and the mechanism of neutralization is the competitive binding of NAbs and S proteins to receptors [33]. Various recombinant monoclonal antibodies (mAbs) are being tested in therapy, with targets to some of the responses caused by SARS-CoV-2 [34,35]. Here, we tested the binding inhibition effect of IgG1 mAb using SMFS (The IgG1 mAb was purchased from ACRObiosystems). The glass substrate was coated with ACE2 using 20 kDa PEG linker. The AFM probes modified with S protein were incubated with mAb and then approached to substrate at a constant speed of 1 μm/s. When the tip contacted with the substrate for 100 ms to react with ACE2 on the surface, it was retracted at the same velocity (Figure 3(A)). The experiments with isotype control antibody IgG1 and without addition of mAb were designed as control. IgG1 was extracellular-signal-related kinase 1and 2 (ERK 1/2), and it is a mouse monoclonal antibody raised against amino acids 325-345 of ERK 1 of rat origin. The force-extension curve was shown in Figure 3(B). We evaluated the neutralization effect of the antibody by measuring the binding probability (BP) of S protein and ACE2 before and after adding the antibody. Compared with the control groups, we observed a significant reduction of the BP when mAb was added (Figure 3(C)). In addition, we studied the inhibition effect of the antibody at single-cell level by measuring the BP between different S proteins and Vero cells with high expression of ACE2 receptor (Figure 3(D)). The representative FECs for the binding of S protein and Vero cell was shown in Figure 3(E). After addition with mAb, lower BP was observed (Figure 3(F)), which in agreement with our previous observation at single-molecule level. The BP of S protein and ACE2 coated substrate is higher than that of S protein and Vero cells. It is possible that the binding event of cell surface and receptor detected by the probe might be interfered by other components on the cell surface. Alsteens’ group have reported that ACE2-derived peptides, 9-O-Acetylated-sialic acid and neutralizing antibodies can have similar inhibitory effect on WT, Alpha, Beta, Gamma, and Kappa variants of SARS-Cov-2 [2,19,20]. Omicron and Delta variants have more infectious and contagious comparing to previous variants [15,36]. Here our results show that mAb can effectively inhibit the binding of S proteins from Delta and Omicron variants BA.1 and BA.5 to ACE2. There have been some neutralization experiments showed the mAb (AM122) have a certain inhibitory and neutralization against SARS-CoV-2 variants Alpha, Beta, Kappa, wild type, and Delta [37,38]. Our data are consistent with the previous tests.

In the treatment and prevention of SARS-CoV-2, there have been many reports on the testing of the neutralizing performance of various antibodies to SARS-CoV-2 and various variants [39,40]. In Omicron spike protein, there exist at least 30 amino acids were mutated, including about 10 at the interface where it interacts with ACE2, and these mutations may be related to Omicron’s higher transmissible and immune escape [41,42]. Federico et al reported a neutralizing antibody STE90-C11 selected from COVID-19 patients could bind to the ACE2-RBD interface and is tolerant to most known RBD mutations in the emerging B.1.525, B.1.526, B.1.1.33, B.1.258, B.1.429/B.1.427, and B.1.617 variants [43]. However, it has slightly reduced affinity to Omicron RBD [41]. Wang et al tested a panel of 17 SARS-CoV-2 monoclonal antibodies (mAbs), and found Omicron could resist seven of eight authorized/approved mAbs, and only the approved antibody S309 was found could retain its neutralizing activity against the original form of Omicron, while it lost more neutralizing activity against Omicron sub-lineages BA.2 and BA.3 [42]. COV2-2196 was showed as similar neutralization against B.1.1.529 with S309 [44]. COV2-2130 could neutralize against BA.2 and BA.3, while lost its neutralizing activity against BA.1 and BA.1.1 [39]. Zhou et al reported the highly potent LY-CoV1404 retain potent neutralizing against all Omicron sub-lineages [39,44]. Therefore, current results have indicated that most neutralizing antibodies induced by wild type SARS-CoV-2 cannot effectively neutralize Omicron, and only sporadic monoclonal antibodies can neutralize Omicron.

### Binding mechanisms of RBD-ACE2 revealed by molecular dynamics simulations

Both SMFS and SCFS experiments showed that, except for Gamma variant, all the variants show higher adhesion force with ACE2 compared with WT, and S protein from Delta variant is the most adhesive to ACE2 protein, while the molecular mechanism is still unclear. Here we use molecular dynamics (MD) simulation to study the molecular mechanism of interaction between ACE2 and RBD of various variants. The RMSD values in Figure S3 reflect that the simulations have reached equilibrium. The interaction residues located at interface of RBD and ACE2 were shown in Figure S4.

**Figure 4.**
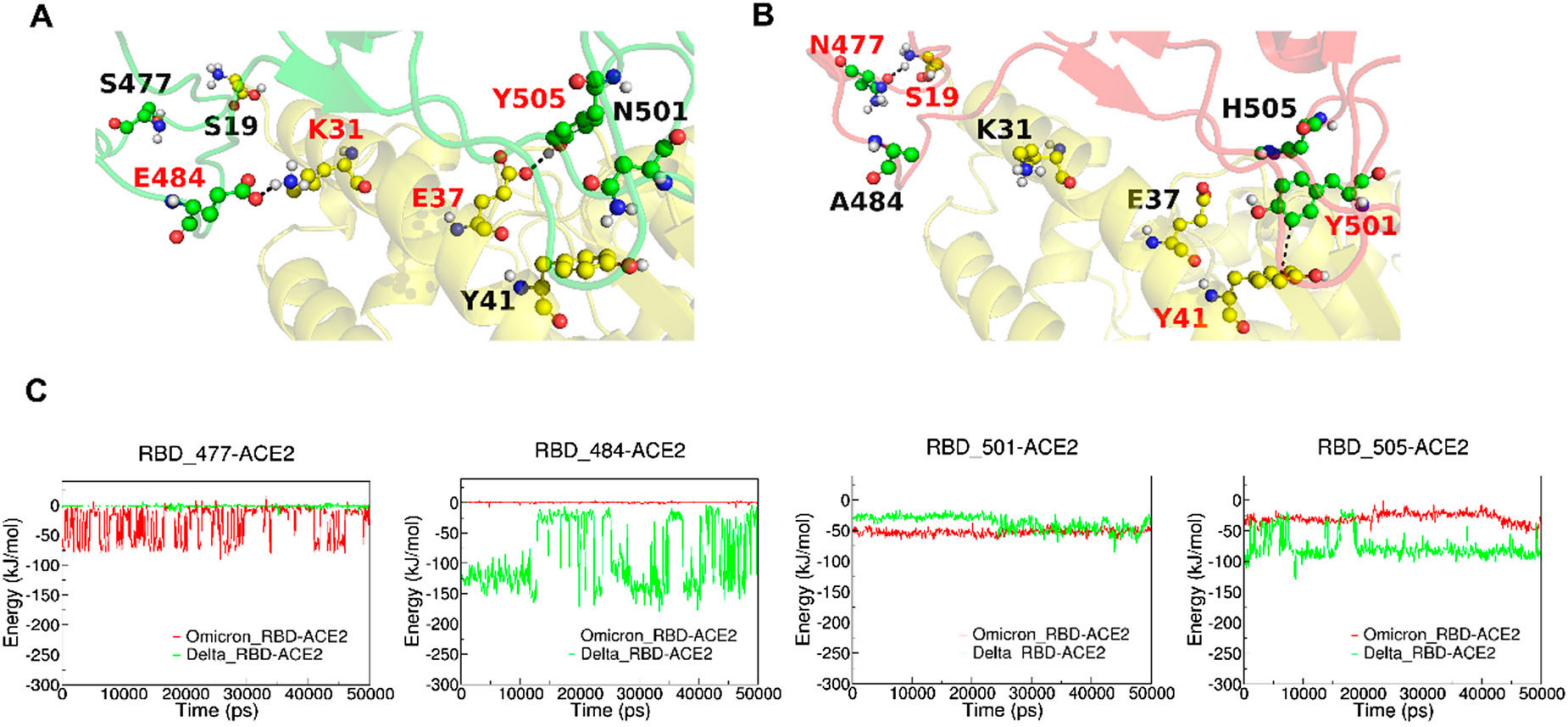
MD simulations of ACE2-RBD complex. (A and B): The interactive interface of ACE2 (yellow) and RBD from Delta (A, green) and Omicron (B, red) variants. (C): Interaction Energy of ACE2 and the individuals of 477, 484, 501 and 505 amino acid residues on RBD from Delta (green) and Omicron (red) Variants.

Our MD simulation results showed that ACE2 and RBD from Delta and Omicron (BA.1) variants have similar interaction energy (Figure S5). The mutations on the RBD were summarized in Table S1. We then analysed the interactions between ACE2 and some key residues (different in Delta and Omicron BA.1 variants) on RBD at the ACE2-RBD interface as shown in Figure 4. As shown in Figure 4(C), we found that K31-E484 and E37-Y505 in the ACE2-RBD (Delta) complex have strong electrostatic interactions (salt bridge and hydrogen bond, respectively), which are absent in ACE2-RBD (Omicron BA.1) complex due to E484A and Y505H mutations, while N501Y and S477N mutations stabilize ACE2-RBD (Omicron BA.1) complex by forming the π-π stacking interaction Y41-Y501 and hydrogen bond S19-N477, respectively.

We analysed the interaction between amino acids located at the interface of RBD and ACE2. Omicron (BA.5) has the most mutations on RBD, and among them, there are many residues located at the interface of RBD and ACE2. The mutations residues 498R, 477N located on RBD formed a new hydron bonding with residues 42Q and S19 on ACE2, respectively. The mutation residue K417N on RBD disappears electrostatic interaction with D30 on ACE2. The N501Y introduced additional π-π interactions with 41Y on ACE2 and a cation π bond with lysine 353 on ACE2, which are possibly increasing the binding force compared with wild type. There exist one mutation N501Y on the RBD of Alpha, like BA.5, and this mutation results in the same additional interactions with ACE2. For the Gamma, three mutated residues were included in the RBD (K417T, E484K, N501Y). Although the N501Y increased the RBD binding with ACE2, the salt bridge interaction between 417 K(RBD) and 30D(ACE2) are disrupted, which may result in a little lower bind force in Gamma with ACE2. There are some studies reported that the K417T could cause the important conformational changes [45]. Delta Plus contain the mutations residues of K417N, L452R, and T478K. We found the interaction between 417 K(RBD) and 30D(ACE2) also disrupted, however the mutation residue 478 K on RBD forms a new hydrogen bonding interaction with 24Q on ACE2. There are some studies reported the L452R and T478K on the RBD could enhance the affinity binding to ACE2 [11,46]. The Alpha, Beta, Omicron, and Gamma contain the same mutation of N501Y on the RBD. Besides, Beta includes mutations of K417N and E484K on the RBD. Although the K417N mutation cause the original electrostatic interaction 417 K(RBD)-30D(ACE2) to disappear, a new hydrogen bonding interaction was generated in 498Q(RBD)-42Q(ACE2) and 493Q(RBD)-35E(ACE2) due to the conformational change of the Beta-ACE2 complex. Similarly, a new hydrogen bonding interaction was generated in 498Q(RBD)-353K(ACE2) and 493Q(RBD)-31K(ACE2) of Kappa result from conformational change. The E484Q in the Kappa RBD plays an important role in binding. The increased long-range Coulomb force interaction after the mutation limits subsequent conformation adjustments during the binding/dissociation of ACE2 and RBD or maybe this evolution appears stable the interface contacts of the termini of the α-helix ACE2 receptor encompassing residues [19,47,48].

**Figure 5.**
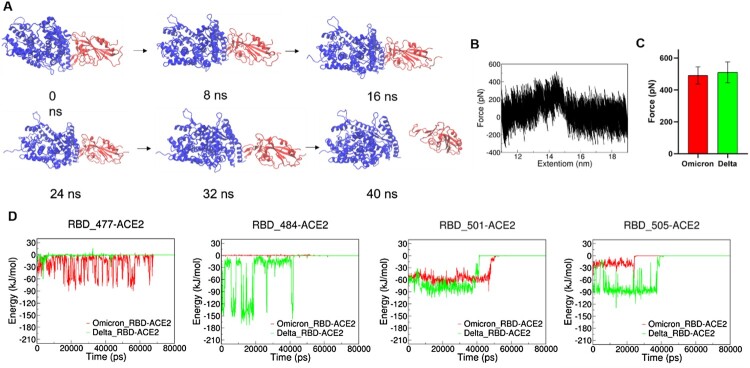
SMD simulation of RBD and ACE complex. (A) Schematics of pulling RBD away from ACE2. (B) Representative force-extension curve of pulling RBD away from ACE2. (C) Unbinding forces of ACE2 and RBD from Delta and Omicron variants. (D) Interaction energy between ACE2 and individual amino acid residues on RBD.

To mimic the force spectroscopy experiments, we performed steered molecular dynamics (SMD) simulations by pulling the RBD away from the ACE2-RBD (Delta and Omicron BA.1 variants) complexes. Figure 5(A) shows the process from the binding state of RBD and ACE2 to the dissociation state and the dissociation occurs in 40 ns. The highest pulling force occurred at ∼31,000 ps and the extension of ∼14.5 nm as shown in Figure 5(B). Our SMD results showed that the ACE2-RBD (Delta) has slightly higher unbinding force (Figure 5(C)), which is consistent with our SMFS experiments. Interactions between ACE2 and key residues on RBD were analysed as shown in Figure 5(D). Similar to our MD results, in the SMD simulations, E484-ACE2 and Y505-ACE2 in the ACE2-RBD (Delta) are more stable upon pulling than A484-ACE2 and H505-ACE2 in ACE2-RBD (Omicron BA.1), while the N477-ACE2 and Y501-ACE2 in ACE2-RBD (Omicron BA.1) are more stable than S477-ACE2 and N501-ACE2 in ACE2-RBD (Delta). We calculated the binding free energy ΔG of RBD and ACE2 in the delta complex by the umbrella sampling method. The results are shown in Figure S6 A, ΔG_delta_ ≈ 42 kJ/mol. The convergence of this potential of mean force (PMF) curve is demonstrated by the umbrella histogram (Figure S6 B), where all centroid distances along the sampling path are covered. Zheng et al reported that N501Y mutation results in higher binding affinity of S protein from Beta variant to ACE2, which is in agreement with our results here [6]. Our SMD process did not capture the additional interactions generated by mutant amino acids L452R and T478K, while there have been reported the mutants L452R and T478K could increase the stability and intra-chain interactions of spike protein, which may result in the higher binding strength to ACE2 [11,46]. Shaimaa et al have showed that the mutations in the RBD of the Delta induced conformational changes in ACE2-E37, which enhanced the electrostatic interactions by the formation of a salt-bridge with SARS-CoV-2-R403 through Molecular Mechanics (MM) and Monte Carlo (MC) sampling [46]. Arunbet et al found that the spike mutants-L452R, T478K, and N501Y have a higher binding affinity to human ACE2 using Protein-protein docking and Molecular mechanics with generalised Born and surface area solvation (MM/GBSA) binding free energy analysis [49].

## Conclusions

Our results suggest S protein from Delta variant has stronger adhesive interaction than S proteins from other variants. At present, the affinity properties of Delta and ACE2 and the neutralizing ability of monoclonal antibody against Delta have been covered in recent reports [37,50], but no study has been found to characterize the adhesion strength of Delta and ACE2 by the method of SMFS. The adhesion force of Omicron variant was not higher and the mutated residues on RBD did not result in stronger interaction with ACE2 when compared with Delta variant. Mutations on RBD from Omicron variant have complex effect on the binding affinity to ACE2. Although Omicron had the largest number of amino acid mutations in RBD, its adhesion to ACE was not the highest. As a new variant, Omicron replaced all variants and became the main popular strain at an absolute speed [15,16], indicating that the adhesion between spike protein and ACE2 was not directly related to the virulence. Many mutations in Omicron may be related to its strong infectivity and immune escape. The E484A and Y505H mutations destabilize the binding, while N501Y and S477N stabilize the binding. It is possible that this adhesion behaviour is related to the stronger infectivity of Omicron BA.1 variant. In addition, we found that the monoclonal antibody produced using WT SARS-Cov-2 can effectively inhibit the binding of S proteins from Delta and Omicron to ACE2 at both single-molecule and cell levels. Our result suggests that although vaccines produced using wild type S protein has showed much lower efficiency to the omicron variant in reality, effective monoclonal antibody can be prepared using wild type S protein against the Delta and Omicron variants by inhibiting the pathogen-host adhesion.

## Methods

*Proteins.* All the SARS-CoV-2 S proteins (Omicron: SPN-C52Z, Omicron: S1N-M122, Delta: SPN-C82Ec, Delta Plus: SPN-C52Ht, Alpha: SPN-C82E5, Beta: SPN-C82E4, Kappa: SPN-C82E7, Gamma: SPN-C82E6 and Wild Type: SPN-C82E9), ACE2 protein (AC2-H52H4) and anti-SARS-CoV-2 Spike RBD neutralizing antibody, Chimeric mAb (AM122), recombinantly expressed from HEK293 cells, were purchased from ACRObiosystems. ERK 1/2 was purchased from Santa Cruz Biotechnology, Inc. Each vial contains 200 μg lgG, kappa light chain in 1.0 ml of PBS with <0.1% sodium azide and 0.1% gelatine.

*Cell culture.* Vero cells with overexpression of ACE2 receptor and HEK293T cells without expression of receptor ACE2 were cultured in Dulbecco modified Eagle medium (DMEM), supplemented with 10% (vol/vol) fetal bovine serum (FBS), 100 U/ml penicillin and 100 μg/ml streptomycin in a humidified atmosphere with 5% CO_2_ at 37°C. Cells were cultured in T25 cell culture flasks and grown to 80–90% for force spectroscopy experiments.

*Functionalization of AFM probes and substrates.* The surface functionalization was performed as described previously [51,52]. Briefly, The MLCT-Bio-DC AFM probes (Bruker) and glass substrates were immersed in 50% isopropanol for 5 min, cleaned in a UV radiation and ozone (UV-O) cleaner and silanized with (3-aminopropyl)-triethoxysilane (APTES). A functional polyethylene glycol (PEG) cross-linker, NHS-PEG-NHS (20 kDa), was used to attach the S protein (0.1 mg/ml) and ACE2 (0.1 mg/ml) to glass substrates and AFM probes, respectively. AFM probes and glass substrates were rinsed with PBS buffer (pH 7.4) for three times and immediately used for force spectroscopy experiments.

*Single-Molecule Force Spectroscopy Experiments.* Atomic force microscope (Nanowizard 4, JPK) was used to acquire the FECs. The experiments were performed in 10 mM PBS buffer (pH 7.4). The spring constant of the cantilever was calculated by measuring its sensitivity and the thermal noise analysis, ranging from 0.04 to 0.06 N/m. The probe modified by S protein approached to substrate at a constant velocity, contacted with the ACE2 modified substrate for 0.1 s, and retracted away from substrate at the same velocity. The FECs recorded were analysed using Igor 6.37 software. The unbinding rate constant and the distance from native state to transition state were estimated through Monte Carlo simulation and Bell-Evans model as described previously [25,53].

*Single-Cell Force Spectroscopy.* Nanowizard 4 equipped with a motorized stage (JPK Instruments) and a pump (FluidFM^®^ MFCS V2), mounted on an inverted optical microscope were used for SCFS experiments. The temperature was set to 37°C by a Petri dish heater (JPK Instruments). The probe is a FluidFM^®^ micropipette with an aperture of 4 μm and initial specified stiffness of 0.3 N/m. Each probe was calibrated before the measurement using the thermal noise analysis. To acquire a single cell to the cantilever, overnight cultivated Vero and HEK293T cells were detached from the culture flask with 0.25% (w/v) trypsin for up to 2 min. Single cells were adsorbed to the probe by applying negative pressure of 200 mbar to the cells. When the single cell was adsorbed to the probe stably, a pressure of 50 mbar was employed to stabilize cell adsorption and avoid cell rupture caused by high pressure.

In the SCFS experiment, the cantilever attached with a single cell was approached to the S protein coated substrate at a velocity of 2 μm/s. After contacting the S protein for 5 s at contact force of 2 nN, the cantilever was retracted at the same speed for 30 μms until the cells were thoroughly separated from the substrate. After one cycle of measurement, the probe with cell absorbed was immersed in 10% (v/v) sodium hypochlorite solution for 2 min, aiming to clean the probe of any remaining cell debris, during which positive pressure of 999 mbar was applied continuously to make the probe excrete cells. The probe is then washed again in water and another fresh cell was absorbed and interacted with the substrate. Adhesion forces were quantified after the FECs were baseline-corrected using JPK data process analysis software. Statistical analysis was done using GraphPad Prism 8.0.2 software.

*Antibody inhibition assays*. The anti-SARS-CoV-2 S protein antibody mAb (AM122), purchased from ACRObiosystems, was obtained through immunizing a mouse with wildtype SARS-CoV-2 Spike S1 protein and then expressed from HEK293 cells. The ACE2 and the S protein were coated to the glass substrate and the AFM probe, respectively. S protein coated probe was incubated with 100 μM antibody mAb for 1 h. Two control groups were designed as follows: one group had no antibody and the other group had the isotype control antibody IgG1 replaced with the same type of antibody. The incubation time and concentration of the control antibody were the same as above. The setpoint force was 200 pN and the probe approached the substrate at a rate of 1 μm/s, contacted with the substrate for 0.1 s, and then retracted at the same speed. The scanning range is 5 × 5 μm, and the sample was scanned using 32 × 32 pixels per line. When studying the binding probability between the S protein and the Vero cells, the set point was set as 450 pN, and the tip was approached the cell at a constant speed of 5 μm/s, contacted with the cell for 400 ms, then retracted to 2 μm at the same speed. The sample was scanned using 32 × 32 pixels per line scanning range is 5 × 5 μm.

*Molecular Dynamics Simulations*. The RBD structures model in complex with ACE2 were taken from the Protein Data Bank (WT PDB: 6m0j; Delta PDB:7w9i; Omicron PDB: 7wbl; Alpha PDB: 7ekf; Gamma PDB: 7ekc; Beta PDB: 7vx4; Kappa PDB: 7vx5;). The complex structure of BA.5(RBD)-ACE2 and Delta Plus(RBD)-ACE2 were designed using Pymol based on Omicron and Delta. Molecular dynamics simulations were carried out using GROMACS 2018.3 [54] with the charmm36m force field [55]. The structures and force fields of Delta and Omicron in complex with ACE2 were obtained by charmm-gui [56]. The system was solvated in a water box using TIP3P model. Counter ions (Na^+^ and Cl^–^) were added to the simulation box to achieve electroneutrality. After neutralizing the complexes, the systems were subjected for energy minimization by adopting the steepest descent energy minimization procedure. The temperature was maintained at 303.15 K and the pressure was maintained at 1 atmosphere. The equilibration of systems was done in the NPT ensemble for 1 ns with backbone constraints. Molecular dynamics (MD) simulation was performed for 50 ns without backbone constraints. Steered molecular dynamics (SMD) simulation was performed by harmonically restraining the position of the C-terminus of ACE2 and pulling on the C-terminus of RBD at a constant pulling speed of 1 Å/ns for 80 ns. Data were analysed by VMD 1.9.3 program, Pymol and LigPlot^+^ 1.4.5.

*Umbrella sampling.* With the potential of mean force (PMF), umbrella sampling simulation provides a robust method to determine ΔG of a protein–protein interaction [57]. In this approach, ΔG is obtained by calculating the difference between the highest and lowest values of the PMF curve after convergence at centre of mass distance. The centroid is defined as the representing average point of the mass. In addition, the “wham utility” of the GROMACS package is used to perform a weighted histogram analysis method (WHAM) to extract PMF values during simulations.

The whole procedure is described as follows. For pulling simulations, the atomic coordinates resulted from the last trajectories of MD simulations, as described in the “Molecular dynamics simulations” section, were used as starting structures. Prior to pulling simulation, energy minimization and the only 200 ps of NPT ensemble were conducted. Subsequently, immobile references were adjusted for pulling simulations via the induction of restraints on RBD-ACE2 proteins. The pull rate of 0.1 nm ns^-1^ (1.0 Å/ns) and the spring constant of 100 kJ mol^-1^ nm^-2^ were used in the pulling of the peptides outside the structures’ centres along the corresponding axis over 8 ns. The final COM distance between peptides and RBD-ACE2 proteins was approximately 19 nm. In the next step, an approximate number of 40 snapshots of pulling simulations’ trajectories were taken to obtain the primary structures for the windows of umbrella sampling. The window spacing was defined as 0.2 nm of COM separation. Afterward, each window was utilized for 20 ns of MD simulation which makes the total umbrella sampling simulation time approximately 800 ns. Ultimately, the PMF curve was produced and the differences of the maximum and minimum values of PMF were used to obtain the ΔG binding for different complexes of proteins_RBD-ACE2 by WHAM utility analyses.

## Supplementary Material

Supplemental Material

## References

[1] Liang K-H, Shih-Han Ko PYC, Chou Y-C, et al. Antibody cocktail effective against variants of SARS-CoV-2. J Biomed Sci. 2021;28:80, doi:10.1186/s12929-021-00777-9. 34814920 PMC8609252

[2] Yang J, Petitjean SJL, Koehler M, et al. Molecular interaction and inhibition of SARS-CoV-2 binding to the ACE2 receptor. Nat Commun. 2020;11:4541, doi:10.1038/s41467-020-18319-6. 32917884 PMC7486399

[3] Li M, Lou F, Fan H. SARS-CoV-2 variants of concern Delta: a great challenge to prevention and control of COVID-19. Signal Transduct Target Ther. 2021;6:349, doi:10.1038/s41392-021-00767-1. 34580279 PMC8475295

[4] Pei P, Qin H, Chen J, et al. Computational design of ultrashort peptide inhibitors of the receptor-binding domain of the SARS-CoV-2 S protein. Brief Bioinform. 2021;22; doi:10.1093/bib/bbab243. 34180984

[5] Zumla A, Chan JFW, Azhar EI, et al. Coronaviruses – drug discovery and therapeutic options. Nat Rev Drug Discov. 2016;15:327–347. 26868298 10.1038/nrd.2015.37 PMC7097181

[6] Tian F, Liang Sun BT, Shi S, et al. N501y mutation of spike protein in SARS-CoV-2 strengthens its 2 binding to receptor ACE2. Elife. 2021;10:e69091. 34414884 10.7554/eLife.69091 PMC8455130

[7] Yan R, Yuanyuan Z, Li Y, et al. Structural basis for the recognition of SARS-CoV-2 by full-length human ACE2. Science. 2020;367:1444–1448. 32132184 10.1126/science.abb2762 PMC7164635

[8] Millet JK, Whittaker GR. Host cell proteases: critical determinants of coronavirus tropism and pathogenesis. Virus Res. 2015;202:120–134. 25445340 10.1016/j.virusres.2014.11.021 PMC4465284

[9] Gu H, Chen Q, Yang G, et al. Adaptation of SARS-CoV-2 in BALB/c mice for testing vaccine efficacy. Science. 2020;369:1603–1607. 32732280 10.1126/science.abc4730 PMC7574913

[10] Yuan M, Huang D, Lee C-CD, et al. Structural and functional ramifcations of antigenic drift in recent SARS-CoV-2 variants. Science. 2021;373:818–823. 34016740 10.1126/science.abh1139 PMC8284396

[11] Motozono C, Toyoda M, Zahradnik J, et al. SARS-CoV-2 spike L452R variant evades cellular immunity and increases infectivity. Cell Host Microbe. 2021;29:1124–1136.e11. 34171266 10.1016/j.chom.2021.06.006 PMC8205251

[12] Liu Z, Van Blargan LA, Bloyet LM, et al. Identifcation of SARS-CoV-2 spike mutations that attenuate monoclonal and serum antibody neutralization. Cell Host Microbe. 2021;29:477–488.e4. 33535027 10.1016/j.chom.2021.01.014 PMC7839837

[13] Chen Y, Chen L, Yin S, et al. The third dose of CoronVac vaccination induces broad and potent adaptive immune responses that recognize SARS-CoV-2 Delta and Omicron variants. Emerg Microbes Infect. 2022;11:1524–1536. doi:10.1080/22221751.2022.2081614. 35608053 PMC9176682

[14] Espenhain L, Funk T, Overvad M, et al. Epidemiological characterisation of the first 785 SARS-CoV-2 Omicron variant cases in Denmark, December 2021. Eurosurveillance. 2021;26; doi:10.2807/1560-7917.Es.2021.26.50.2101146. PMC8728489 34915977

[15] Tuekprakhon A, Nutalai R, Dijokaite-Guraliuc A, et al. Antibody escape of SARS-CoV-2 Omicron BA.4 and BA.5 from vaccine and BA.1 serum. Cell. 2022;185:2422–2433.e13. doi:10.1016/j.cell.2022.06.005. 35772405 PMC9181312

[16] Dejnirattisai W, Huo J, Zhou D, et al. SARS-CoV-2 Omicron-B.1.1.529 leads to widespread escape from neutralizing antibody responses. Cell. 2022;185:467–484.e15. doi:10.1016/j.cell.2021.12.046. 35081335 PMC8723827

[17] Han P, Li L, Liu S, et al. Receptor binding and complex structures of human ACE2 to spike RBD from Omicron and Delta SARS-CoV-2. Cell. 2022;185:630–640.e10. doi:10.1016/j.cell.2022.01.001. 35093192 PMC8733278

[18] Ravichandran S, Coyle EM, Klenow L, et al. Antibody signature induced by SARS-CoV-2 spike protein immunogens in rabbits. Sci Transl Med. 2020;12:eabc3539. 32513867 10.1126/scitranslmed.abc3539 PMC7286538

[19] Koehler M, Ray A, Moreira RA, et al. Molecular insights into receptor binding energetics and neutralization of SARS-CoV-2 variants. Nat Commun. 2021;12:6977. doi:10.1038/s41467-021-27325-1. 34848718 PMC8633007

[20] Petitjean SJL, Chen W, Koehler M, et al. Multivalent 9-O-acetylated-sialic acid glycoclusters as potent inhibitors for SARS-CoV-2 infection. Nat Commun. 2022;13:2564, doi:10.1038/s41467-022-30313-8. 35538121 PMC9091252

[21] Wang Y, Liu C, Zhang C, et al. Structural basis for SARS-CoV-2 Delta variant recognition of ACE2 receptor and broadly neutralizing antibodies. Nat Commun. 2022;13:871. doi:10.1038/s41467-022-28528-w. 35169135 PMC8847413

[22] Tai L, Zhu G, Yang M, et al. Nanometer-resolution in situ structure of the SARS-CoV-2 postfusion spike protein. Proc Natl Acad Sci. 2021;118:e2112703118. doi:10.1073/pnas.2112703118. 34782481 PMC8640741

[23] Lv Z, Deng Y-Q, Ye Q, et al. Structural basis for neutralization of SARS-CoV-2 and SARS-CoV by a potent therapeutic antibody. Science. 2020;369:1505–1509. doi:10.1126/science.abc5881. 32703908 PMC7402622

[24] Zhang X, Kou X, Zhang W, et al. Identification of the new type of G-Quadruplex with multiple vacant sites in human telomeric DNA. CCS Chemistry. 2022;4:3023–3035. doi:10.31635/ccschem.021.202101436.

[25] He C, Hu C, Hu X, et al. Direct observation of the reversible two-state unfolding and refolding of an α/β protein by single-molecule atomic force microscopy. Angew Chemie – Int Ed. 2015;54:9921–9925. 10.1002/anie.201502938 26136291

[26] Moghaddar M, Radman R, Macreadie I. Severity, pathogenicity and transmissibility of Delta and Lambda variants of SARS-CoV-2, toxicity of spike protein and possibilities for future prevention of COVID-19. Microorganisms. 2021;9:2167; doi:10.3390/microorganisms9102167. 34683488 PMC8540532

[27] Müller DJ, Helenius J, Alsteens D, et al. Force probing surfaces of living cells to molecular resolution. Nat Chem Biol. 2009;5:383–390. 19448607 10.1038/nchembio.181

[28] Maynard SA, Gelmi A, Stacey C, et al. Nanoscale molecular quantification of stem cell–hydrogel interactions. ACS Nano. 2020;14:17321–17332. doi:10.1021/acsnano.0c07428. PMC7760213 33215498

[29] Helenius J, Heisenberg CP, Gaub HE, et al. Single-cell force spectroscopy. J Cell Sci. 2008;121:1785–1791. 18492792 10.1242/jcs.030999

[30] Puckert C, Tomaskovik-Crook E, Gambhir S, et al. Molecular interactions and forces of adhesion between single human neural stem cells and gelatin methacrylate hydrogels of varying stiffness. Acta Biomater. 2020;106:156–169. 32084598 10.1016/j.actbio.2020.02.023

[31] Bharadwaj M, Strohmeyer N, Colo GP, et al. αV-class integrins exert dual roles on α5β1 integrins to strengthen adhesion to fibronectin. Nat Commun. 2017;8:14348. doi:10.1038/ncomms14348. 28128308 PMC5290147

[32] Spoerri PM, Zhiqi Sun NS, Fässler R, et al. Protease-activated receptor signalling initiates α5β1-integrin-mediated adhesion in non-haematopoietic cells. Nat Mater. 2020;19:218–226. doi:10.1038/s41563-019-0580-4. 31959953

[33] Klasse PJ, Moore JP. Antibodies to SARS-CoV-2 and their potential for therapeutic passive immunization. Elife. 2020;9. doi:10.7554/eLife.57877. PMC7311167 32573433

[34] Prompetchara E, Tanapat Palaga CK. Immune responses in COVID-19 and potential vaccines lessons learned from SARS and MERS epidemic. Asian Pac J Allergy Immunol. 2020;38:1–9. doi:10.12932/AP-200220-0772. 32105090

[35] Valdez-Cruz NA, Clara Espitia EGH, et al. Integrative overview of antibodies against SARS CoV 2 and their possible applications in COVID 19 prophylaxis and treatment. Microb Cell Factories. 2021;20:32. doi:10.1186/s12934-021-01526-1. PMC8061467 33888152

[36] Jhun H, Park HY, Hisham Y, et al. SARS-CoV-2 Delta (B.1.617.2) variant: a unique T478K mutation in receptor binding motif (RBM) of Spike Gene. Immune Netw. 2021;21:e32, doi:10.4110/in.2021.21.e32. 34796036 PMC8568914

[37] Zhang JZ, Yeh H-W, Walls AC., et al. Thermodynamically coupled biosensors for detecting neutralizing antibodies against SARS-CoV-2 variants. Nat Biotechnol. 2022;40:1336–1340. doi:10.1038/s41587-022-01280-8. 35484405 PMC9463068

[38] Kozminsky M, Carey TR, Sohn LL. DNA-directed patterning for versatile validation and characterization of a lipid-based nanoparticle model of SARS-CoV-2. Adv Sci. 2021;8:2101166. 10.1002/advs.202101166 PMC8646752 34672117

[39] Ai J, Wang X, He X, et al. Antibody evasion of SARS-CoV-2 Omicron BA.1, BA.1.1, BA.2, and BA.3 sub-lineages. Cell Host Microbe. 2022;30:1077–1083.e4. doi:10.1016/j.chom.2022.05.001. 35594867 PMC9080084

[40] Hansen J, Baum A, Pascal KE, et al. Studies in humanized mice and convalescent humans yield a SARS-CoV-2 antibody cocktail. Science. 2020;369:1010–1014. doi:10.1126/science.abd0827. 32540901 PMC7299284

[41] Omotuyi O, Olubiyi O, Nash O, et al. SARS-CoV-2 Omicron spike glycoprotein receptor binding domain exhibits super-binder ability with ACE2 but not convalescent monoclonal antibody. Comput Biol Med. 2022;142:105226. doi:10.1016/j.compbiomed.2022.105226. 35066447 PMC8739363

[42] Wang X, Zhao X, Song J, et al. Homologous or heterologous booster of inactivated vaccine reduces SARS-CoV-2 Omicron variant escape from neutralizing antibodies. Emerg Microbes Infect. 2022;11:477–481. doi:10.1080/22221751.2022.2030200. 35034583 PMC8820826

[43] Bertoglio F, Fühner V, Ruschig M, et al. A SARS-CoV-2 neutralizing antibody selected from COVID-19 patients binds to the ACE2-RBD interface and is tolerant to most known RBD mutations. Cell Reports. 2021;36:109433. doi:10.1016/j.celrep.2021.109433. 34273271 PMC8260561

[44] Zhou T, Wang L, Misasi J, et al. Structural basis for potent antibody neutralization of SARS-CoV-2 variants including B.1.1.529. Science. 2022;376:eabn8897. doi:10.1126/science.abn8897. 35324257 PMC9580340

[45] Wang P, Nair MS, Liu L, et al. Antibody resistance of SARS-CoV-2 variants B.1.351 and B.1.1.7. Nature. 2021;593:130–135. doi:10.1038/s41586-021-03398-2. 33684923

[46] Goher SS, Ali F, Amin M. The Delta variant mutations in the receptor binding domain of SARS-CoV-2 show enhanced electrostatic interactions with the ACE2. Med Drug Discov. 2022;13:100114, doi:10.1016/j.medidd.2021.100114. PMC8650763 34901826

[47] Rankl C, Kienberger F, Wildling L, et al. Multiple receptors involved in human rhinovirus attachment to live cells. Proc Natl Acad Sci. 2008;105:17778–17783. doi:10.1073/pnas.0806451105. 18997008 PMC2584741

[48] Qiao B, Olvera de la Cruz M. Enhanced binding of SARS-CoV-2 spike protein to receptor by distal polybasic cleavage sites. ACS Nano. 2020;14:10616–10623. doi:10.1021/acsnano.0c04798. 32806067

[49] Gurung AB, Ali MA, Lee J, et al. Structural and functional insights into the major mutations of SARS-CoV-2 spike RBD and its interaction with human ACE2 receptor. J King Saud Univ – Sci. 2022;34:101773, doi:10.1016/j.jksus.2021.101773. 34955621 PMC8686452

[50] Kim S, Liu Y, Lei Z, et al. Differential interactions between human ACE2 and spike RBD of SARS-CoV-2 variants of concern. bioRxiv: The Preprint Server for Biology. 2021; doi:10.1101/2021.07.23.453598. PMC8672429 34856802

[51] Shi S, Wang Z, Deng Y, et al. Combination of click chemistry and enzymatic ligation for stable and efficient protein immobilization for single-molecule force spectroscopy. CCS Chem. 2022;4:598–604. doi:10.31635/ccschem.021.202100779.

[52] Zhang X, Chen J, Li E, et al. Ultrahigh adhesion force between silica-binding peptide SB7 and glass substrate studied by single-molecule force spectroscopy and molecular dynamic simulation. Front Chem. 2020;8:600918, doi:10.3389/fchem.2020.600918. 33330393 PMC7729015

[53] Bell GI. Models for the specific adhesion of cells to cells. Science. 1978;200:618–627. doi:10.1126/science.347575. 347575

[54] Abraham MJ, Murtola T, Schulz R, et al. GROMACS: high performance molecular simulations through multi-level parallelism from laptops to supercomputers. SoftwareX. 2015;1–2:19–25.

[55] Huang J, Rauscher S, Nawrocki G, et al. CHARMM36m: an improved force field for folded and intrinsically disordered proteins. Nat Methods. 2017;14:71–73. doi:10.1038/nmeth.4067. 27819658 PMC5199616

[56] Lee J, Cheng X, Swails JM, et al. CHARMM-GUI input generator for NAMD, GROMACS, AMBER, OpenMM, and CHARMM/OpenMM simulations using the CHARMM36 additive force field. J Chem Theory Comput. 2016;12:405–413. 26631602 10.1021/acs.jctc.5b00935 PMC4712441

[57] Lemkul JA, Bevan DR. Assessing the stability of Alzheimer’s amyloid protofibrils using molecular dynamics. J Phys Chem B. 2010;114:1652–1660. 20055378 10.1021/jp9110794

