# Supplementary material for: Pathogen-host adhesion between SARS-CoV-2 spike proteins from different variants and human ACE2 studied at single-molecule and single-cell levels
Source: Emerg Microbes Infect. 2022 Nov 4;11(1):2658–69. doi: 10.1080/22221751.2022.2128887 (PMC9639500; doi:10.1080/22221751.2022.2128887)
Supplement: Supplemental Material [file TEMI_A_2128887_SM6881.docx]

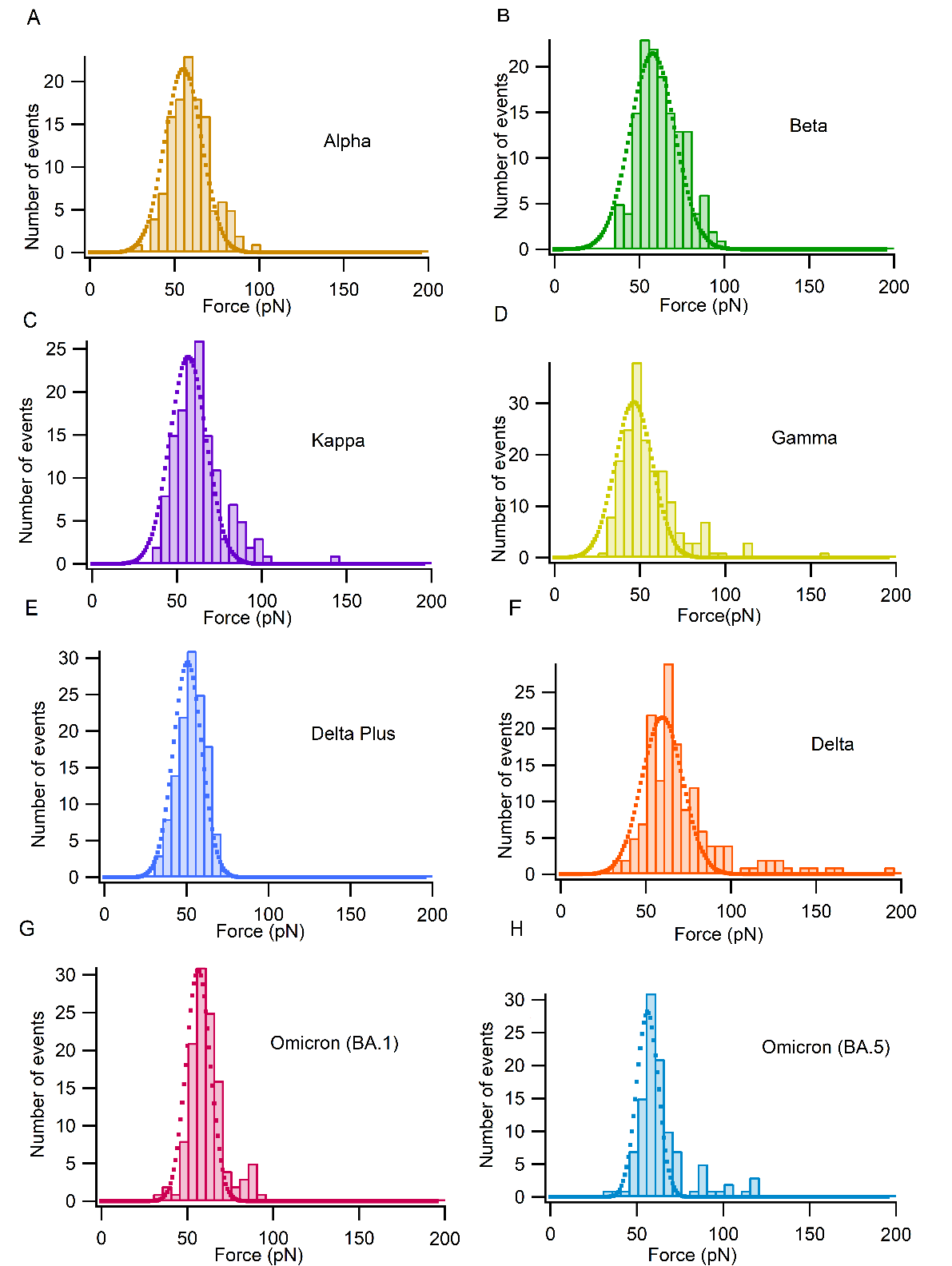


I


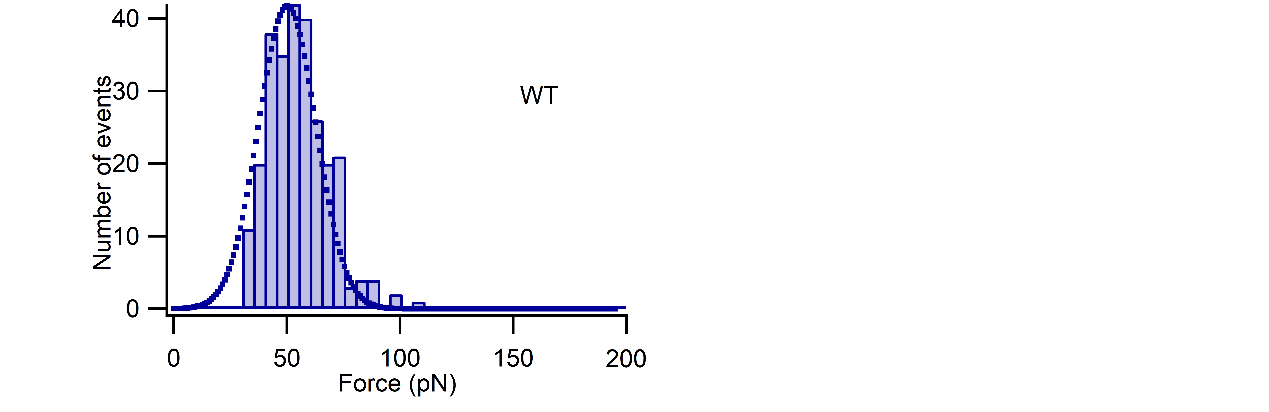


**Figure S1.** Representative histograms of rupture force distribution for the interaction between ACE2 and different S proteins. Dashed lines represent the results of gaussian fitting.


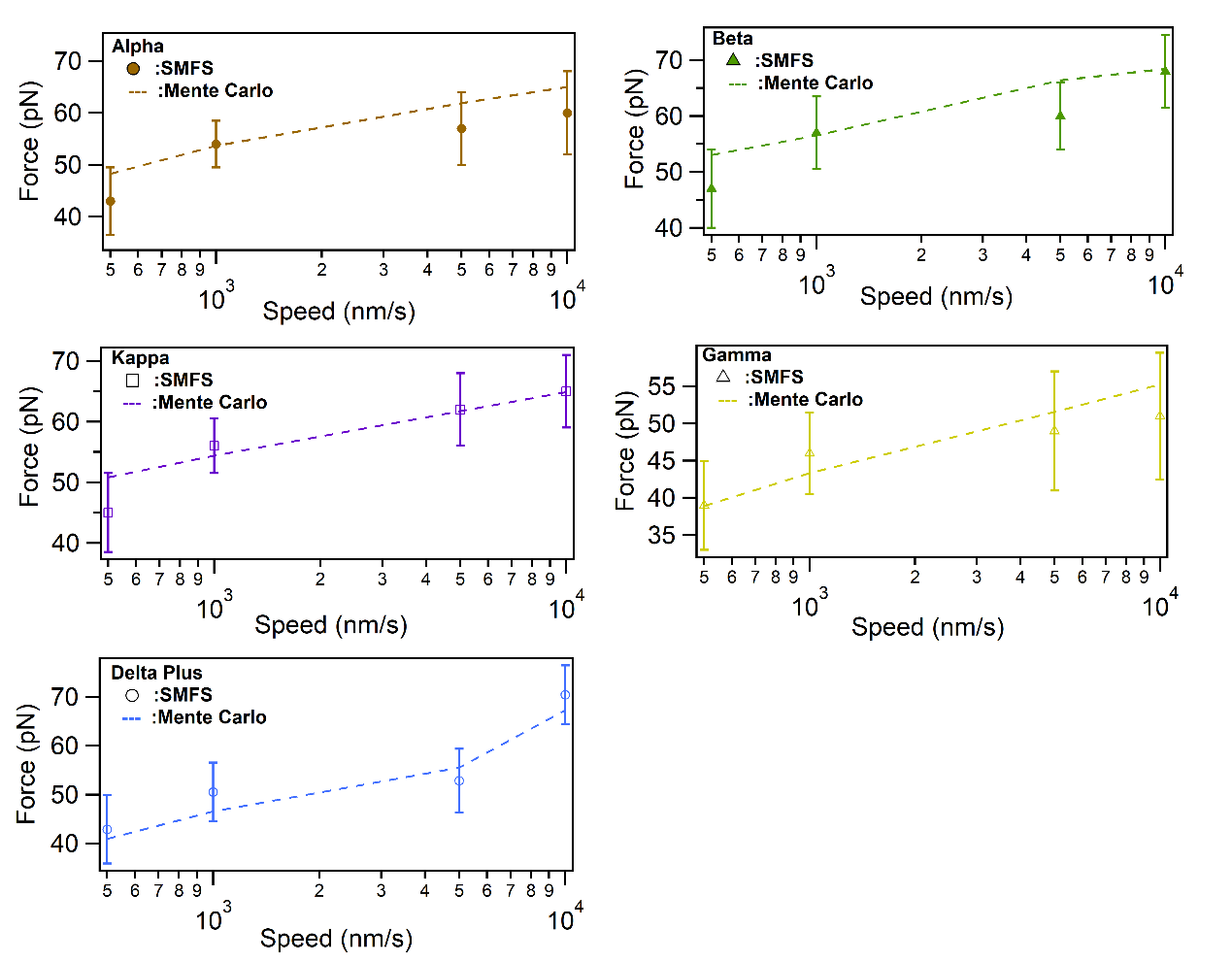


**Figure S2.** Pulling speed dependency of adhesive forces and Monte Carlo simulations for extracting the kinetic parameter. Solid dots represent experimental data and dotted lines represent simulated results.

**
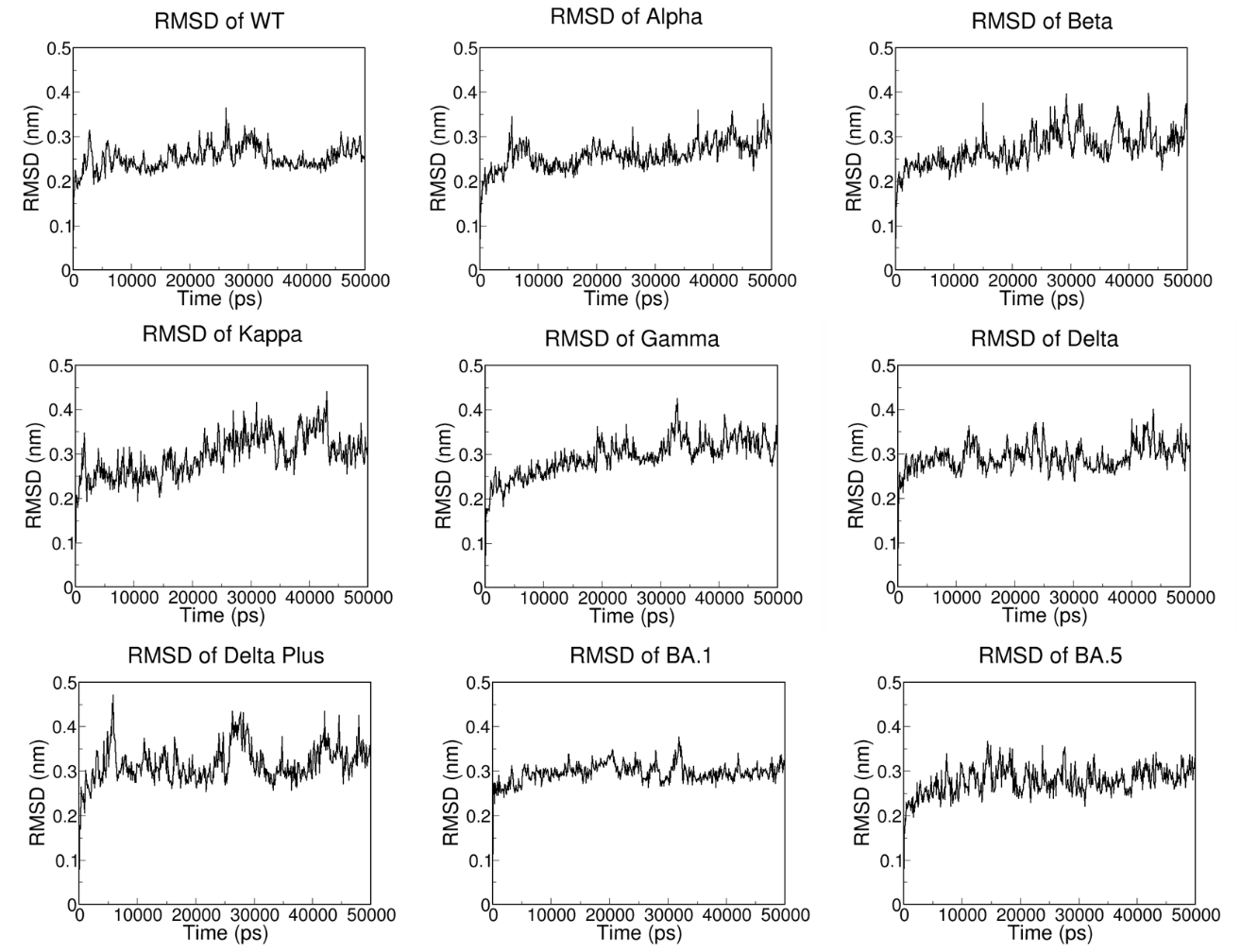
**

**Figure S3.** RMSD values of the molecular dynamics simulations.


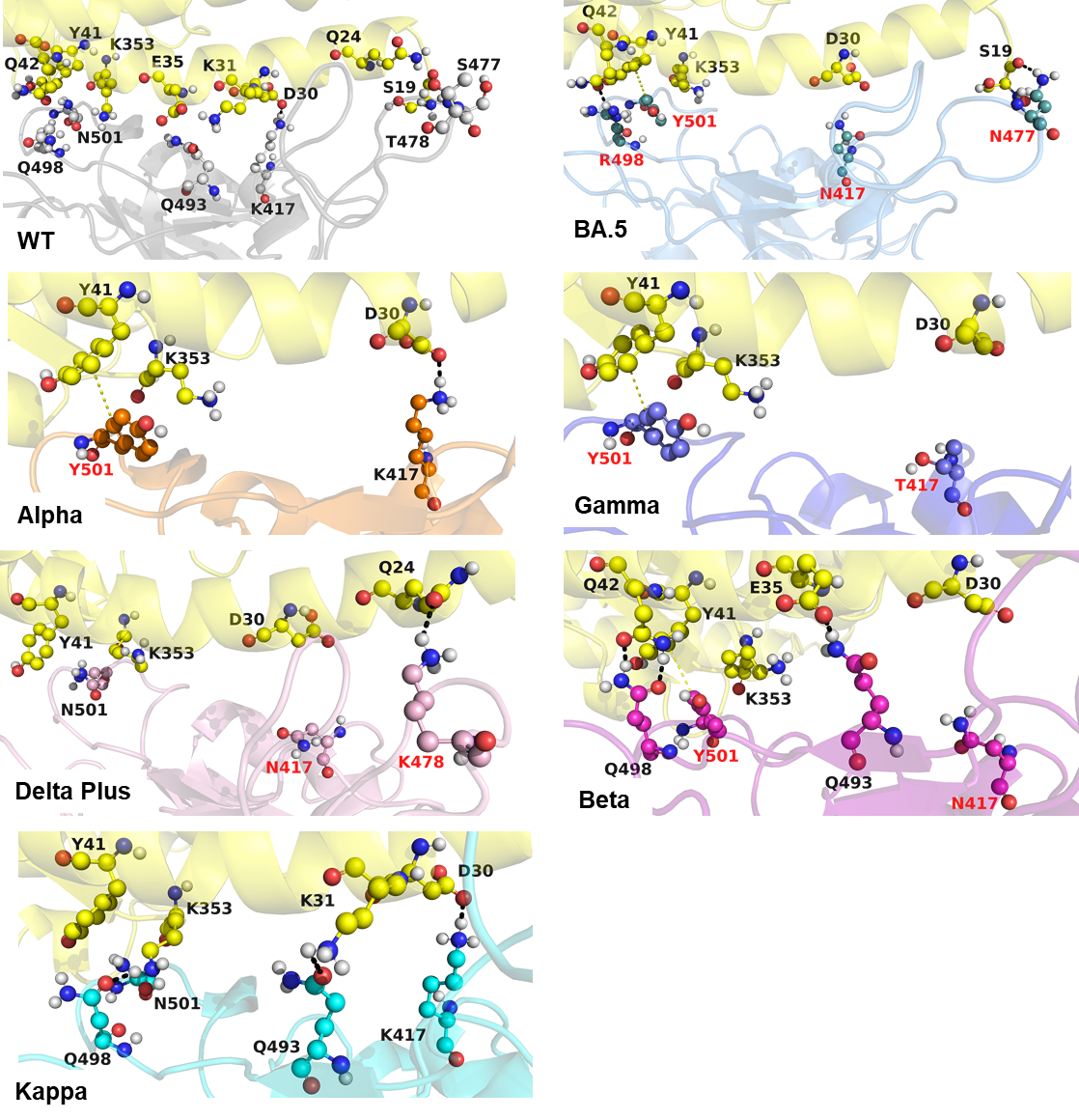


**Figure S4.** MD simulation of the different RBD-ACE2 complexes of Wild Type (yellow) and VoCs Alpha (orange), Beta (purple), Gamma (blue), Kappa (cyan), Delta Plus (pink) and Omicron (BA.5). Residues involved in the interaction between RBDs and ACE2 are represented by sphere. The black dashed lines represent hydrogen bond interactions and the yellow dashed lines represent π-π interactions.


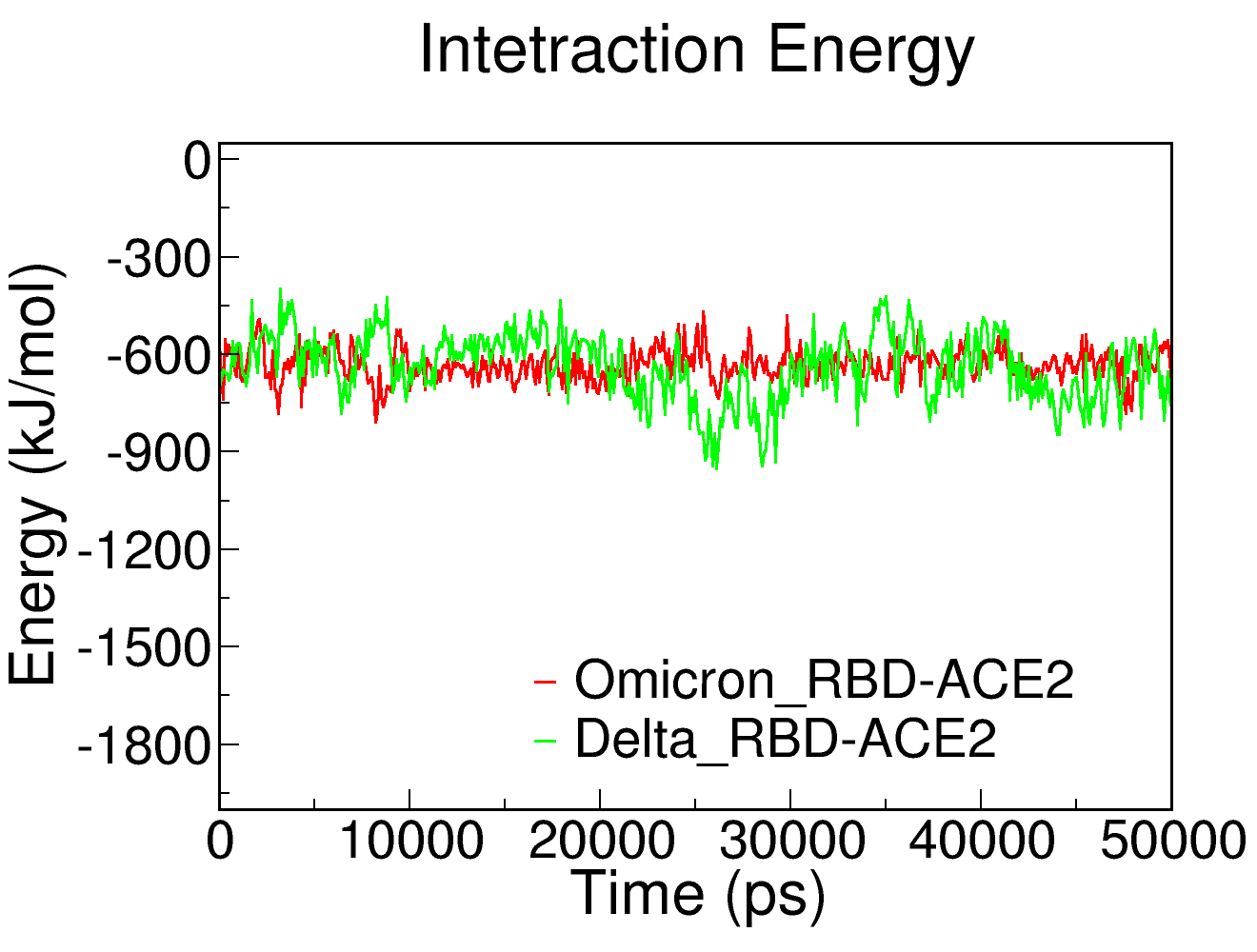


**Figure S5.** Energy diagrams of Omicron (BA.1) and Delta in molecular dynamics simulations.

**
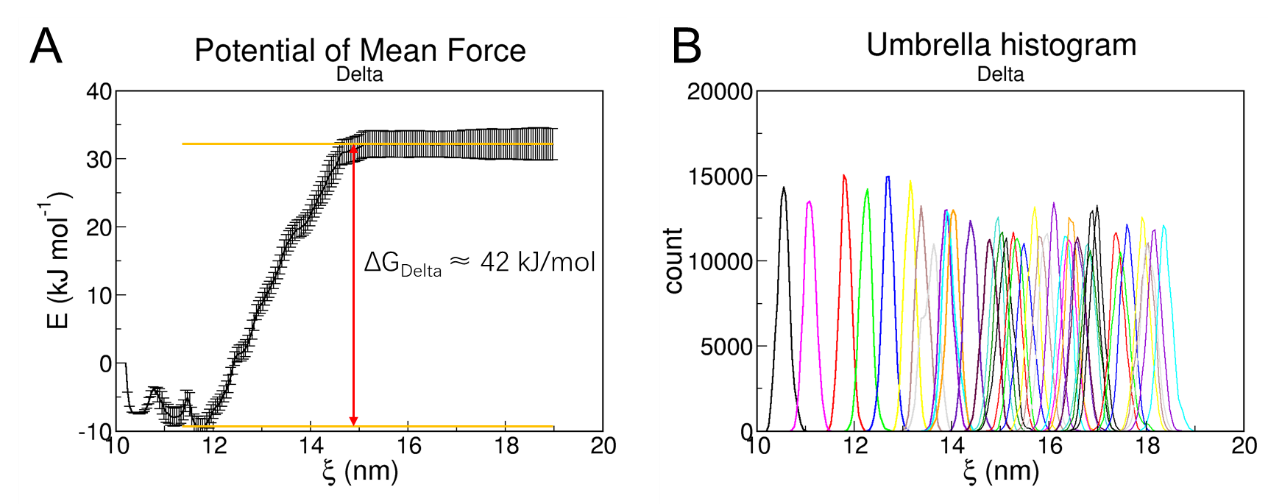
**

**Figure S6.** PMF of Delta in umbrella sampling. **A.**PMF curve of Delta. **B.** The umbrella histograms of Delta.

**Table S1.** Mutated amino acids in different variants of RBD

| RBD | Mutations |
| --- | --- |
| Omicron  (BA.1) | K417N, G446S, S477N, T478K, E484A, Q493R, G496S, Q498R, N501Y, Y505H |
| Omicron  (BA.5) | K417N, L452R, S477N, T478K, E484A, F486V, Q498R, N501Y, Y505H |
| Delta | L452R, T478K |
| Delta Plus | K417N, L452R, T478K |
| Alpha | N501Y |
| Gamma | K417T, E484K, N501Y |
| Kappa | E484Q |
| Beta | K417N, E484K, N501Y |
